# Hyperpigmentation of the hard palate mucosa in a patient with chronic myeloid leukaemia taking imatinib

**DOI:** 10.1186/s40902-017-0136-y

**Published:** 2017-12-05

**Authors:** Gian Paolo Bombeccari, Umberto Garagiola, Francesco Pallotti, Margherita Rossi, Massimo Porrini, Aldo Bruno Giannì, Francesco Spadari

**Affiliations:** 1 Maxillo-Facial and Dental Unit, Fondazione Ca’ Granda IRCCS Ospedale Maggiore Policlinico, University of Milan, Via Commenda 10, 20122 Milan, Italy; 2 Department of Biomedical, Surgical and Dental Sciences, University of Milan, Milan, Italy; 3 Unit of Anatomical Pathology, Fondazione IRCCS Ca’ Granda Ospedale Maggiore Policlinico, University of Milan, Via Commenda 10, 20122 Milan, Italy

**Keywords:** Chronic myeloid leukaemia, Oral melanosis, Drug-induced oral reactions, Oral pigmentation, Mucosal pigmentation

## Abstract

**Background:**

Imatinib mesylate is an inhibitor of the tyrosine kinase Bcr–Abl and a first-line treatment for Philadelphia chromosome-positive chronic myeloid leukaemia (CML). Dermatological side effects include superficial oedema, pustular eruption, lichenoid reactions, erythroderma, and skin rash. Depigmentation of the skin and/or mucosa is uncommon, and hyperpigmentation is rare.

**Case presentation:**

We present the case of a 63-year-old Caucasian male with widespread hyperpigmentation of the hard palate associated with a 9-year history of imatinib therapy to treat CML. He did not complain of any symptoms. Clinical examination did not reveal any abnormal pigmentation of the skin or other region of the oral mucosa. He did not smoke cigarettes or drink alcohol. His medication regimen was a proton pump inhibitor, a beta-blocker, cardioaspirin, atorvastatin, and imatinib 400 mg/day. Histopathologically, melanin and haemosiderin deposits were evident in the lamina propria. The lesion persisted, with no clinical change, through several follow-ups. We reviewed the literature to explore the possible relationship between oral hyperpigmentation and long-term imatinib mesylate treatment.

**Conclusions:**

We diagnosed oral pigmentation associated with imatinib intake based on the medical history and clinical features of the pigmented macules. Oral pigmentation may have a variety of causes, and differential diagnosis requires nodal analysis. Clinicians should be aware of possible oral mucosal hyperpigmentation in patients taking imatinib mesylate. Such pigmentation is benign and no treatment is needed, but surveillance is advisable.

## Background

Pigmentation of the oral mucosa associated with overproduction of melanin is relatively common and may involve any region of the oral cavity. The prevalence varies by geographical region and ethnicity. A cross-sectional study of 1275 Jordanian subjects found that 30.2% exhibited oral pigmentation [1]. In Sweden, such lesions are found in about 10% of the population [2]. The differential diagnosis includes physiological and environmental causes, as well as manifestations of systemic disease [3]. Drug-induced pigmentation constitutes 10–20% of all cases of acquired hyperpigmentation and should be considered during diagnosis, especially in elderly patients on multidrug therapy [4]. The aetiology of drug-induced pigmentation varies with the causative drug. One or more of three potential pathways may be involved: these are deposition of the drug per se or a metabolite thereof, stimulation of melanin production, and bacterial metabolism of the drug, alone or in combination [5]. The colour ranges from brown (associated with the use of oral contraceptives) to blue–black (often associated with hydroxychloroquine treatment) [5, 6].

Imatinib mesylate (Gleevec®; Novartis, Basel, Switzerland), a tyrosine kinase inhibitor targeting the Bcr–Abl protein, is a first-line treatment for Philadelphia chromosome-positive CML [7]. The dermatological side effects include superficial oedema and skin rash (the most frequent side effects), pustular and/or lichenoid eruptions, erythroderma, graft-versus-host-like disease, and small-vessel vasculitis [8–10]. Hypopigmentation of the skin and/or mucosa is an uncommon side effect [11]. Intraoral side effects are unusual and, in a few cases, have included lichenoid reactions [12–14] and dental pigmentation [15–17]. Rarely, hyperpigmentation of the hard palate has been observed, presumably related to drug intake [3, 18–23]. Here, we describe a case of widespread hyperpigmentation of the hard palate mucosa associated with long-term imatinib treatment of a CML patient.

## Case presentation

**Fig. 1:**
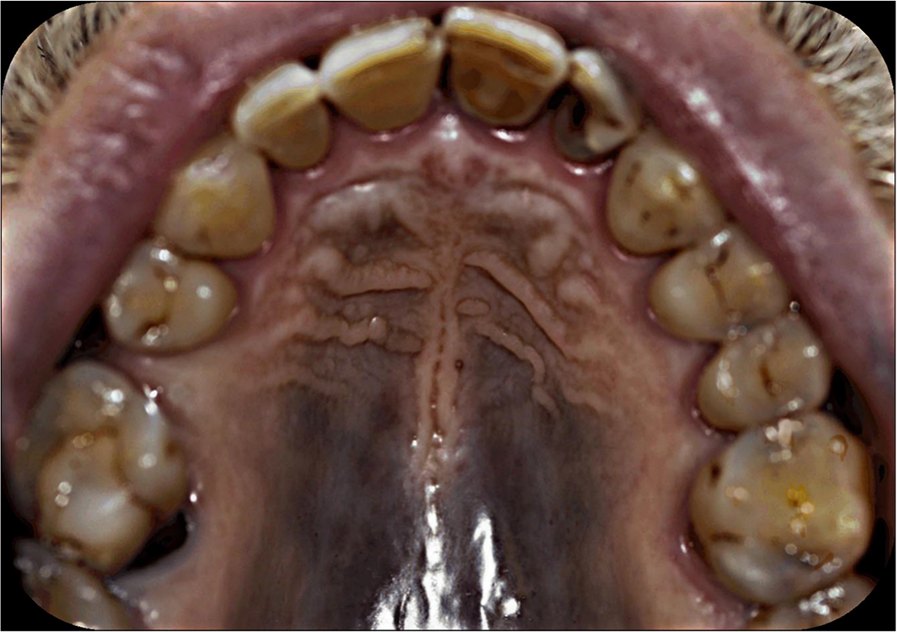
An extensive blue–grey pigmented lesion of the hard palate mucosa evident on clinical examination

In January 2016, a 63-year-old Caucasian male was referred to us for evaluation of painless grey–blue hyperpigmentation of the hard palate, noted by his dentist during a routine dental examination (Fig. 1). His medical history included hypertension, hyperlipidaemia, and CML diagnosed about 10 years prior. His medication regimen was a proton pump inhibitor (20 mg/day), a beta-blocker (50 mg/day), cardioaspirin (100 mg/day), atorvastatin (20 mg/day), and imatinib (400 mg/day). He had been taking imatinib for about 9 years. He had never taken hydroxyurea, minocycline, or any anti-malarial agent. Clinical examination revealed no abnormal pigmentation of the skin or other region of the oral mucosa. He denied smoking and alcohol consumption. We scheduled a complete blood count test and screening for Addison’s disease. No serological abnormalities were evident. Under local anaesthesia, we performed a 3-mm incisional punch biopsy. The histopathological report and medical history were consistent with drug-induced palatal hyperpigmentation. We diagnosed mucosal pigmentation associated with imatinib therapy, thus excluding other environmental, physiological, and pathological causes (Table 1).

**Table 1:** Conditions associated with mucosal pigmentation that should be considered during the differential diagnosis of oral melanosis [1, 6, 25–27]

Environmental causes	
Smoking-associated melanosis	
Heavy metal pigmentation due to metallic deposit	
Dental amalgam tattoos	
Drug-induced pigmentation	
Physiological causes	
Physiological ethnic and/or racial pigmentation	
Labial melanotic macule	
Oral melanocytic nevi	
Pathological causes	
Post-inflammatory deposits of melanin	
Peutz–Jeghers syndrome	
AIDS	
Hemochromatosis	
Addison’s disease	
Laugier–Hunziker disease	
Oral melanoacanthoma	
Pseudo-ochronosis	
Bandler’s syndrome	
McCune–Albright syndrome	
Cowden syndrome	
Neurofibromatosis	
Riehl’s melanosis	
LAMB syndrome (Carney complex)	
Polyostotic fibrous dysplasia syndrome	
LEOPARD syndrome	
Hyperthyroidism	
Nelson’s syndrome	
Melanosis associated with melanoma	

### Histopathological findings

**Fig. 2:**
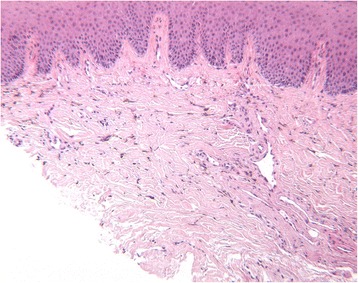
The lamina propria of the oral mucosa contained brown pigment scattered between collagen fibres and in the cytoplasm of macrophages (H&E ×10)

**Fig. 3:**
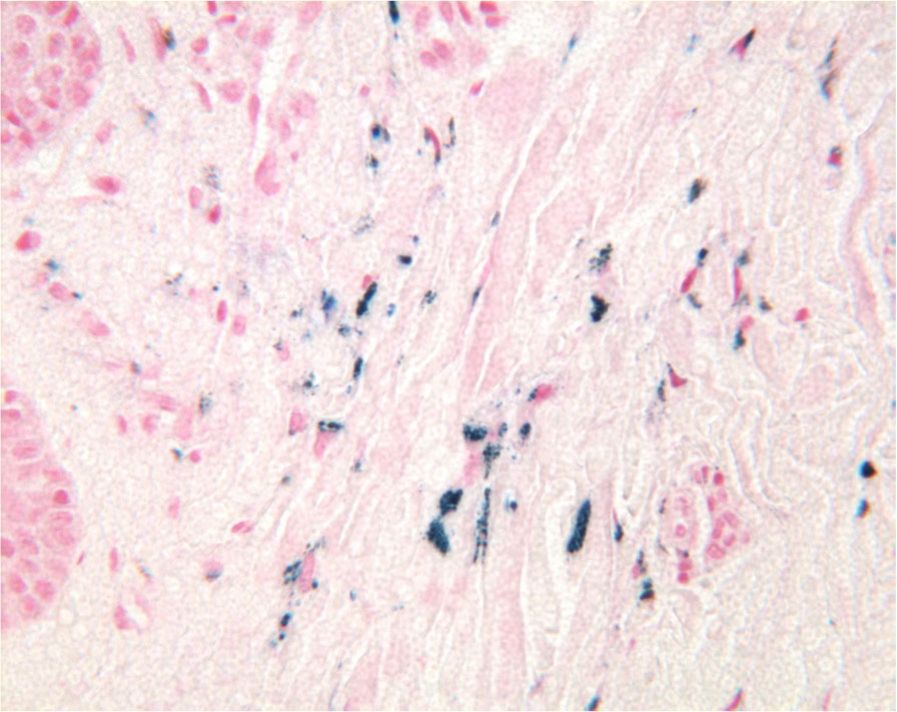
The lamina propria of the oral mucosa contained blue-staining spherical particles that included iron (Perl’s stain, ×40)

**Fig. 4:**
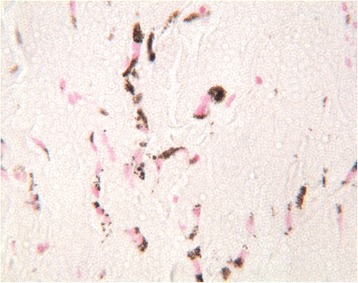
The lamina propria of the oral mucosa contained brown-staining spherical particles that included melanin (Fontana–Masson staining, ×40)

Histopathological examination revealed a non-inflamed palatal mucosa with pigment-containing histiocytes in the mucous membrane (Fig. 2). Immunohistochemically, both haemosiderin (Perl’s Prussian blue staining) and melanin (Fontana–Masson staining) were detected (Figs. 3 and 4).

### Outcome and follow-ups

At the 6-month follow-up, neck ultrasonography did not reveal any swollen lymph node. We took close-up colour photographs of the lesion to confirm the absence of any morphological change. In May 2017, palatal hyperpigmentation was still evident, and the clinical appearance was unchanged, but he reported no symptoms.

### Discussion

Diagnostic considerations when encountering oral melanosis should include physiological, pathological, and environmental variables. Physiological oral melanosis is usually localised to the gingival and buccal mucosa and is bilateral and symmetrical, brownish in colour, and clinically more common among dark-skinned populations [1]. Oral melanotic macules present as well-circumscribed brown-to-black flat lesions, mainly on the lower vermilion. The pathogenesis of physiological melanotic macules remains controversial; both reactive and genetic factors may be involved [6]. Oral nevi typically appear as solitary brown-to-black mucosal macules, mainly on the palate and buccal mucosa. Although the pathogenesis of nevi remains unknown, it has been suggested that the lesions are benign neoplasms. No malignant transformation of oral nevi has yet been reported, and no evidence points to an increased risk of oral melanoma in affected subjects [24]. Notably, palatal melanosis must be differentiated from an oral melanoma, which may present as an asymptomatic brown-to-grey-black macula with irregular borders. Further, an oral melanoma grows rapidly and exhibits ulcerative evolution with bleeding and pain [25]. Several systemic diseases, including Addison’s disease, Peutz–Jeghers syndrome, McCune–Albright syndrome, Cowden syndrome, neurofibromatosis, acquired immunodeficiency syndrome, haemochromatosis, and hyperthyroidism, as well as uncommon conditions such as Nelson’s syndrome, polyostotic fibrous dysplasia syndrome, Laugier–Hunziker syndrome, and the Carney complex, may feature oral melanotic macules [1, 26, 27]. Melanosis associated with these conditions is due to increased levels of melanin within the basal cell layer, attributable to incontinent melanophages in the lamina propria, in the absence of iron deposits and bleeding [3, 28]. Oral pigmentation induced by smoking (smoker’s melanosis) may be associated with the effects of components of tobacco on oral melanocytes [29]. It has been hypothesised that stimulation of melanin production may be a protective reaction of the oral mucosa, associated with detoxification of polycyclic amines and benzopyrenes, thus being a side effect of tobacco use [30]. Post-inflammatory melanin deposits scattered throughout the oral connective tissue are frequently observed in patients with chronic inflammatory diseases such as oral lichen planus, pemphigoid, and pemphigus [31]. Hyperpigmentation following inflammation may be caused by an increase in melanogenesis triggered by cytokines and reactive oxygen species, which induce melanocyte activity and the proliferation of dendritic cells, and increase tyrosinase activity [31, 32].

A history of occupational or environmental exposure to heavy metals and clinical signs of metal toxicity help to identify pigmentations of the oral mucosa. Heavy metals such as bismuth, lead, copper, arsenic, gold, copper, cobalt, chromium, silver, mercury, and magnesium can induce the development of a bluish-black line, the so-called Burton’s line, along the gingival margin, the thickness of which is proportional to the extent of gingival inflammation [31]. In some cases, however, the hard palate mucosa adjacent to amalgam dental fillings develops blue–grey macules, termed the “amalgam tattoo.” Histologically, the amalgam tattoo presents as discrete dark granules or fragments, usually surrounding collagen bundles and blood vessels, associated with low-level infiltrations of inflammatory cells [33]. The aetiology of medication-associated oral pigmentation may be related to the use of drugs that induce melanin formation. These include clofazimine used to treat leprosy, anti-malarials such as quinine, and immunomodulatory agents. In patients on hormonal therapy, conjugated oestrogens can lower the serum cortisol concentration by stimulating adrenocorticotropic hormone (ACTH) production. Notably, oral hyperpigmentation induced by anti-malarials, minocycline, and imatinib often involves the mucosa of the hard palate [18]. Histopathologically, imatinib-induced oral pigmentation usually presents as spherical pigmented melanin bundles in the lamina propria, with no sign of inflammation or haemorrhage [3, 18–20, 23].

Of the 15 cases published in the English language literature, eight reported the histopathological features, i.e. deposits of melanin and/or haemosiderin in the lamina propria. Of these, four described co-existing melanin and haemosiderin deposits. Our findings are consistent with those of the cited reports; both Fontana–Masson staining for melanin and Perl’s Prussian blue staining for haemosiderin were positive (Table 2).

**Table 2:** Summary of previous case reports on oral mucosal pigmentation associated with imatinib therapy

Author(s), year	Duration of treatment with imatinib	Dosage (mg/dose)	Age and sex of patient	Site(s) affected	Histological findings	Condition treated
Singh and Bakhshi 2007 [15]	4 years	300	13 F	Gingivae, teeth	Clinical diagnosis only	CML
Lewis, 2009 [18]	Unknown	800	70 M	Palate	Melanin and haemosiderin deposits in lamina propria	CML
Mcpherson et al. 2009 [38]	6 years	Unknown	59 F	Gingivae, toes, fingernails	Clinical diagnosis only	CML
Wong et al. 2011 [19]	3 months	Unknown	43 F	Palate	Melanin deposits in lamina propria	CML
Mattsson et al. 2011 [20]	5 years 5 years 5 years	400 400 400	66 F 66 F 64 F	Palate Palate Palate	Melanin deposits in lamina propria Clinical diagnosis only	Metastatic gastrointestinal stromal tumour CML CML
Li et al. 2012 [3]	4 years 10 years 5 years	400 400 400	64 M 53 M 29 F	Palate Palate Palate	Melanin and haemosiderin deposits in lamina proria	CML CML Pelvic fibromatosis
Resende et al. 2012 [21]	5 years	600	38 M	Palate, nose, earlobes	Clinical diagnosis only	Post-haematopoietic stem Cells transplant
Roeker and Wolanskyj 2014 [22]	6 years	Unknown	65 F	Palate	Clinical diagnosis only	CML
Song and Kang 2014 [23]	Unknown	Unknown	58 M	Palate, nose	Clinical diagnosis only	CML
Lyne et al. 2015 [24]	13 years	400–600	58 F	Palate	Haemosiderin deposits in lamina propria	CML
Romeo et al. 2015 [39]	11 years	400	72 F	Palate	Brown spherical bodies located within the lamina propria	CML

*CML* chronic myeloid leukaemia

The pathophysiological mechanism of mucocutaneous pigmentation induced by imatinib remains unclear. Imatinib targets the ATP-binding site of the Bcr–Abl tyrosine kinase and also inhibits the actions of other tyrosine kinases, including platelet-derived growth factor receptor-b, C-kit, and C-ABL [17]. C-kit is a transmembrane growth factor expressed in basal skin cells, melanocytes, epithelial cells of the breast, and mast cells, stimulation of which leads to activation (followed by the rapid degradation) of microphthalmia transcription factor (MITF); in turn, this transactivates the promoter of the tyrosinase pigmentation gene of melanocytes [9]. It has been suggested that imatinib inhibits ligand binding to specific receptors on the surfaces of human melanocytes, reducing cellular activity and thus commonly triggering hypopigmentation [10]. However, imatinib may rarely cause hyperpigmentation of the skin and/or mucosae; a metabolite of the drug may chelate iron and melanin, as do minocycline and anti-malarial drugs [3]. Currently, it is not known why the mucosa of the hard palate is the tissue invariably affected by hyperpigmentation. However, the palate contains a large number of mucosal melanocytes [34] in which imatinib metabolites accumulate. Also, C-kit signalling may play a role in oral hyperpigmentation, and indeed, C-kit is widely expressed in mesenchymal cells of the human oral cavity, including dental pulp cells and gingival fibroblasts [35]. In addition, the cases of oral hyperpigmentation reported to date do not appear to be drug dose-dependent (Table 2). Only a few oral mucosal hyperpigmentation cases caused by administration of imatinib mesylate to treat haematological malignancies have been reported. Hence, it remains speculative to suggest that imatinib mesylate may directly influence melanocyte C-kit signalling in the oral mucosa, activating melanogenesis. It is possible that genetic and/or other factors are also involved in the development of oral melanotic maculae. Finally, the time of onset of CML may be relevant; sometimes, patients are treated initially with hydroxyurea, which may also cause mucocutaneous hyperpigmentation and melanonychia [36–39].

## Conclusions

The diagnosis of imatinib-associated oral pigmentation requires a thorough history-taking and clinical examination of the melanotic maculae. Medical and dental practitioners should be aware of possible oral mucosal hyperpigmentation in patients taking imatinib mesylate. The hyperpigmented lesions are benign; no treatment is required. However, annual follow-up is advisable to monitor changes in morphology or colour over time.

## Abbreviation

ACTH: Adrenocorticotropic hormone
CML: Chronic myeloid leukaemia
MITF: Microphthalmia transcription factor
